# Essential Oil from *Melaleuca leucadendra*: Antimicrobial, Antikinetoplastid, Antiproliferative and Cytotoxic Assessment

**DOI:** 10.3390/molecules25235514

**Published:** 2020-11-25

**Authors:** Lianet Monzote, Alexander M. Scherbakov, Ramón Scull, Prabodh Satyal, Paul Cos, Andrey E. Shchekotikhin, Lars Gille, William N. Setzer

**Affiliations:** 1 Parasitology Department, Institute of Tropical Medicine “Pedro Kouri”, 10400 Havana, Cuba; 2 Department of Experimental Tumor Biology, Blokhin N.N. National Medical Research Center of Oncology, 24 Kashirskoye sh., Moscow 115522, Russia; alex.scherbakov@gmail.com; 3 Department of Pharmacy, Institute of Pharmacy and Food, Havana University, La Coronela, La Lisa, 13600 Havana, Cuba; rscull@ifal.uh.cu; 4 Aromatic Plant Research Center, 230 N 1200 E, Suite 100, Lehi, UT 84043, USA; psatyal@aromaticplant.org; 5 Laboratory for Microbiology, Parasitology and Hygiene (LMPH), Faculty of Pharmaceutical, Biomedical and Veterinary Sciences, University of Antwerp, 2610 Antwerp, Belgium; paul.cos@uantwerpen.be; 6 Laboratory of Chemical Transformations of Antibiotics, Gause Institute of New Antibiotics, 11 B. Pirogovskaya St., Moscow 119021, Russia; shchekotikhin@mail.ru; 7 Department of Biomedical Sciences, Institute of Pharmacology and Toxicology, University of Veterinary Medicine, Veterinärplatz 1, 1210 Vienna, Austria; Lars.Gille@vetmeduni.ac.at; 8 Department of Chemistry, University of Alabama in Huntsville, Huntsville, AL 35899, USA

**Keywords:** *Melaleuca leucadendra*, essential oil, protozoa, *Leishmania*, cancer cells, cytotoxicity, BALB/c

## Abstract

Essential oils (EOs) are known for their use in cosmetics, food industries, and traditional medicine. This study presents the chemical composition and therapeutic properties against kinetoplastid and eukaryotic cells of the EO from *Melaleuca* *leucadendra* (L.) L. (Myrtaceae). Forty-five compounds were identified in the oil by GC-MS, containing a major component the 1,8-cineole (61%). The EO inhibits the growth of *Leishmania amazonensis* and *Trypanosoma brucei* at IC_50_ values <10 μg/mL. However, 1,8 cineole was not the main compound responsible for the activity. Against malignant (22Rv1, MCF-7, EFO-21, including resistant sublines MCF-7/Rap and MCF-7/4OHTAMO) and non-malignant (MCF-10A, J774A.1 and peritoneal macrophage) cells, IC_50_ values from 55 to 98 μg/mL and from 94 to 144 μg/mL were obtained, respectively. However, no activity was observed on *Staphylococcus aureus*, *Enterococcus faecalis*, *Escherichia coli*, *Pseudomonas aeruginosa*, *Aspergillus niger*, *Candida parapsilosis*, *Microsporum canis*, or *Trypanosoma cruzi*. The EO was able to control the lesion size and parasite burden in the model of cutaneous leishmaniasis in BALB/c mice caused by *L. amazonensis* compared to untreated animals (*p* < 0.05) and similar with those treated with Glucantime^®^ (*p* > 0.05). This work constitutes the first evidence of antiproliferative potentialities of EO from *M. leucadendra* growing in Cuba and could promote further preclinical investigations to confirm the medical value of this plant, in particular for leishmaniasis treatment.

## 1. Introduction

Plant-derived compounds as an alternative therapy for microbial infections have been established to be one of the most auspicious sources to develop new therapeutic alternatives. During the last decades, an accumulating interest in the screening of plant-derived products has been appreciated considering their availability and safety when compared with synthetic compounds [1]. Among these, essential oils (EOs) constitute an important source of biologically active compounds, which are complex mixtures mostly constituted of secondary metabolites. More than 3000 EOs have been identified or commercialized, due to their frequent use in cosmetics and flavors, as well as in the food industries, like spices, or to prepare beverages. They are also widely known for their use in traditional medicine as antibacterial, insecticidal, fungicidal, nematicidal, herbicidal, antioxidant, and anti-inflammatory agents [2,3].

In particular, antiproliferative potentialities of EOs have been demonstrated, including actions against parasitic protozoans [4] and malignant cell lines [5,6]. To continue our search for new bioactive natural products from Cuban plants, in this study, we have focused on the EO from *Melaleuca leucadendra* (L.) L. (EO-ML) of the Myrtaceae.

**Figure 1:**
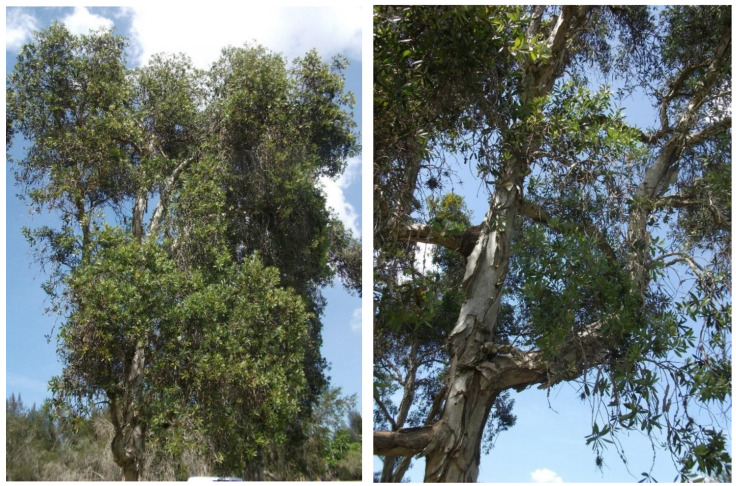
Photographs of *Melaleuca leucadendra* plant cultivated in National Botanic Garden, Havana, Cuba (Picture taken by the authors during the collection of the plant).

*Melaleuca* species are tall shrubs and small trees having a height of up to 7 m with a bushy crown and papery bark. Leaves are usually hairless, 10–35 mm long and about 1 mm wide, while the phyllotaxis of leaves is scattered to whorled. The leaves have prominent glands enriched with aromatic oil [7]. In general, a long history of the medicinal use of this genus is known, mainly because of their broad-spectrum antimicrobial activity [8]. In particular, *M. leucadendra* (Figure 1) has been widely grown in various parts of the world and shows different biological properties. For example, the bark and leaves are used in folk medicine as tranquilizing, sedating, evil-dispelling, and pain-relieving agents [9,10]. Other pharmacological effects have been reported, including antioxidant, anti-inflammatory [11], and antimicrobial [12,13] activities. In addition, in vitro antimicrobial profiling of an ethanol extract from *M. leucadendra* grown in Cuba was performed, exhibiting inhibitory activity against *Microsporum canis*, *Staphylococcus aureus*, *Plasmodium falciparum*, *Trypanosoma cruzi*, *T. brucei*, *Leishmania infantum* [14], and *L. amazonensis* [15].

Based on previous information, this work presents the studies of EO-ML growing in Cuba: (i) analysis of the chemical composition by gas-chromatography coupled with a mass spectrometric detector (GC-MS); (ii) a general antiproliferative in vitro assessment against kinetoplastid parasites (*L. amazonensis*, *T. cruzi*, and *T. brucei*), malignant cell lines: Human prostate carcinoma cell line (22Rv1) and human breast cancer (MCF-7), and non-malignant murine macrophages: Continuous culture using the cell line J774A.1 and primary non-growing macrophage from the peritoneum of BALB/c mice (PMM); (iii) in vitro evaluation of the main compound of EO-ML on more susceptible cultures; and (iv) effects of EO-ML on experimental cutaneous leishmaniasis (CL) in BALB/c mice caused by *L. amazonensis*.

## 2. Results and Discussion

The studied EO-ML presented 45 compounds (Table 1), which constituted 99.9% of the composition and were represented by monoterpene hydrocarbons (10.5%), oxygenated monoterpenoids (79.2%), sesquiterpene hydrocarbons (0.2%), oxygenated sesquiterpenoids (9.6%), benzenoids (0.3%) and others (0.1%). The main components were 1,8-cineole or eucalyptol with 61%, which is a characteristic chemotype of *M. leucadendra* as demonstrated Brophy et al. [16] and An et al. [17]. In addition, other compounds at lower concentrations were identified, such as α-terpineol (15.6%), viridiflorol (7.9%), limonene (4.8%) α- and β-pinene (2.7 and 1.2%, respectively) and terpinen-4-ol (1.2%), which have also been previously reported for EO-ML [15,16].

A review of the literature revealed variation in qualitative and quantitative chemical compositions of EO-ML from plants collected in Cuba, depending on their location. In this sense, Pino et al. reported that the analysis of the aerial parts from *M. leucadendra* collected in Matanzas Province showed also 1,8-cineole (43.0%) as the main compound [18]; while the major component was viridiflorol (28.2%) in samples from Pinar del Rio Province [19]. In parallel, other reports showed the 1,8-cineole chemotype from plants growing in Brazil [20] and Egypt [13] with 48.7% and 64.3% 1,8-cineole, respectively. Nevertheless, other chemotypes have also been documented based on phenylpropanoids (namely eugenol methyl ether and (*E*)-*iso*-eugenol methyl ether) or nerolidol chemotypes [16,17].

**Figure 2:**
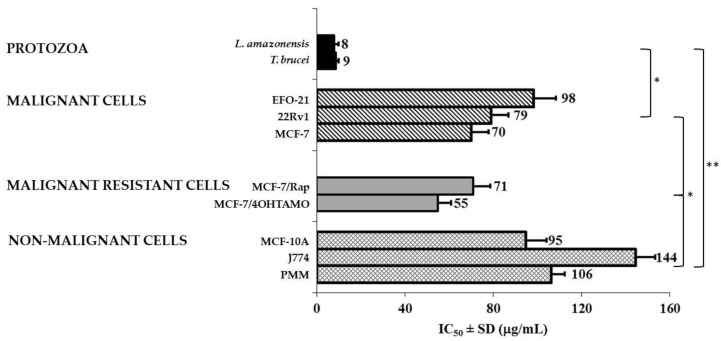
In vitro antitrypanosomatidae, antiproliferative activity, and cytotoxic effects of essential oil extracted by hydrodistillation from *Melaleuca leucadendra* collected in National Botanic Garden, Havana, Cuba. Statistical differences with *p* < 0.05 (*) and *p* < 0.01 (**) compared to control cells.

Although the promising biological activities of EO-ML have been known for decades, herein we compared for the first time the activity of the same EO obtained from plants cultivated in Cuba against different prokaryotic (Gram-positive bacteria: *Staphylococcus aureus*, *Enterococcus faecalis*, Gram-negative bacteria: *Escherichia coli*, *Pseudomonas aeruginosa*, and fungi: *Aspergillus niger*, *Candida parapsilosis*, *Microsporum canis*) and eukaryotic (kinetoplastid parasites: *Trypanosoma* and *Leishmania*, malignant: EFO-21, MCF-7, and 22Rv1, resistant malignant sublines: MCF-7/Rap and MCF-7/4OHTAMO, as well as non-malignant continuous cultures: MCF-10A and J774.A1, and primary macrophages from the peritoneum of BALB/c mice (PMM)). Then, antimicrobial screening of EO-ML shown that this product did not affect bacterial or fungal growth at the maximal tested concentration (500 μg/mL); except against dermatophyte *M. canis* with a bordered activity (MIC 125 μg/mL). However, regarding the parasitic protozoa, EO-ML demonstrated activity against *L. amazonensis* and *T. brucei* with IC_50_ values <10 μg/mL (Figure 2); although no effect was observed against *T. cruzi*. In addition, the antiproliferative effect on malignant cells was also appreciated, which were statistically smaller (*p* < 0.05) than antiprotozoal activities. Nevertheless, similar IC_50_ values (*p* > 0.05) against malignant cells and resistant lines were obtained. Finally, the cytotoxicity assay against non-malignant cells revealed IC_50_ values >94 μg/mL, which generated selectivity indices >11-fold compared with protozoal parasites (*L. amazonensis* and *T. brucei*) and approximately 1 or 2-fold with respect to malignant cell lines.

Although no activity on *T. cruzi* was observed, a general biological activity against eukaryotic cells was appreciated for EO-ML. In particular, a higher sensitivity of kinetoplastid parasites than mammalian cells was found. Recently, Luna et al. [4] demonstrated that in the absence of new effective drugs against *Trypanosoma* and *Leishmania*, many studies demanded the use of EOs. In particular, da Silva et al. [21] reviewed the potential activity of EOs from 35 plant species against *L. amazonensis*, of which 45.7% had an IC_50_ < 10 μg/mL. Our results, together with reports in the literature, corroborate the antikinetoplastid potential of EOs and could stimulate the development of these natural products as a source of new phytotherapeutics for leishmaniasis and trypanosomiasis treatment.

In addition, the EO-ML showed inhibitory activity against tested malignant cell lines, including resistant sublines, with IC_50_ values ranging from 55 to 98 μg/mL. The anticancer potential of various EOs has attracted a great deal of interest, and extensive research has been carried out to characterize the anticancer activity, the molecular mechanisms, the chemopreventive potential, and the chemotherapeutic application of these products [5]. Although the American National Cancer Institute considers natural products, including EOs, to be active with IC_50_ values below 30 μg/mL [22], the antiproliferative property of the EO-ML could be in consideration, particularly against resistant sublines. Currently, either intrinsic or acquired resistance during the course of treatment is a limiting factor in successful cancer chemotherapy and constitutes one of the major challenges for cancer chemotherapy [23]. In this regard, the most interesting result of the studied EO was its capability to limit the growth of resistant sublines, as well as other malignant susceptible cell lines, which may lead to further studies to prevent or attack proliferation of resistant malignant cells as a needed therapeutic strategy.

Follow-up studies were carried out on kinetoplastid parasites that showed higher susceptibility to EO-ML. In this case, the evaluation of 1,8 cineole against *L. amazonensis* and *T. brucei* parasites was performed. It is an important strategy for its applicability and can facilitate the search for the biological mechanism by which the oil may act [24]. In addition, although the oils are complex mixtures, a major compound may have a greater influence on the observed biological activity [24]. In this study, however, 1,8 cineole showed IC_50_ values of 68.3 ± 3.4 µg/mL (0.44 ± 0.02 mM) and 30.3 ± 1.5 µg/mL (0.19 ± 0.01 mM) against *L. amazonensis* and *T. brucei*, respectively. Furthermore, no cytotoxicity was observed against non-malignant macrophages at 200 µg/mL. Thus, 1,8 cineole is not the main agent responsible for the antiprotozoal activity of EO-ML. Nibret and Wink reported an IC_50_ of 83.1 µg/mL for this compound against *T. brucei* [25]. On the other hand, Machado et al. [26] documented that this pure compound did not display an important inhibitory activity on *L. infantum*, which represented 58.6% of the *Thymus capitellatus* Hoffmanns & Link EO; while Santana et al. [27] and Camargos et al. [28] showed that 1,8-cineole was able to inhibit the growth of *L. amazonensis* with IC_50_ values of 48.4 μg/mL (0.3 mM) and 724 μg/mL (4.7 mM) against amastigotes and promastigotes, respectively. Therefore, the activity of EO-ML could result from the complex interactions between their constituents. In some cases, these interactions may lead to antagonistic or synergistic effects that contribute to the biological activity of EOs, and even minor components of EOs can play a critical role in these effects. There have been a few studies concerning antikinetoplastid actions of the minor EO-ML constituents. For example, some authors showed IC_50_ values of: 4.2 μg/mL for limonene, 1.0 μg/mL for α-pinene [29], 47.4 μg/mL for β-pinene [30] and 0.02 μg/mL for terpinen-4-ol [31] against *T. brucei*; as well as 37.9 μg/mL for limonene [32] 105 μg/mL for α-terpineol [28] and 37 μg/mL for α-pinene [27] against *L. amazonensis*. Although the concentrations of these compounds were less than 1,8-cineole, the presence of known components with antikinetoplastid activity in the oil could, therefore, account for the inhibitory effect found for EO-ML. Nevertheless, further studies with minor components or compound combinations are needed to identify the compound(s) responsible for the effects of EO-ML.

Note that the previous report of this plant against bacteria, fungi, and parasites was conducted with an aqueous ethanolic extract [14,15]. Although the chemical composition of this extract was not reported, the different procedures for solvent extraction and EO hydrodistillation will result in very different chemical compositions, marked mainly in relatively polar, non-volatile in the ethanol extract, and volatile non-polar compounds in the EO [33]. Nevertheless, antiparasitic activities were displayed by both the ethanol extract and the EO, which strongly suggests the potentialities of *M. leucadendra*.

It is concluded, therefore, that the oil exhibited comparable susceptibilities against both *L. amazonensis* and *T. brucei*, and presented higher in-vitro activity and selectivity than the pure main component. However, Caridha et al. [34] highlighted the need for new treatments for CL, due to current treatments showing poor justification through clinical trials and sub-optimal effectiveness [35,36]. In particular, against *L. amazonensis*, the EO-ML displayed an IC_50_ of 8 μg/mL and a selectivity index = 18 (with respect to J774) and 13 (with respect to PMM), which are in concordance with international criteria related to the development of natural products for cutaneous species, i.e., (i) classification as highly active with IC_50_ < 10 μg/mL [37] and (ii) a selectivity index >5 [34]. In the Neotropics, *L. amazonensis* is considered to be one of the most important species that causes cutaneous leishmaniasis (CL). In addition, nearly 1% of all CL cases can develop an anergic diffuse CL infection, which is characterized by massive dermal infiltrates and presents clinical, immunological, parasitological, anatomopathological, and therapeutic responses different from other CL forms. This clinical presentation is chronic with frequent relapses due to non-response to conventional treatment [26,38]. Thus, the effect of EO-ML was evaluated in the model of experimental CL caused by *L. amazonensis* in BALB/c mice.

**Figure 3:**
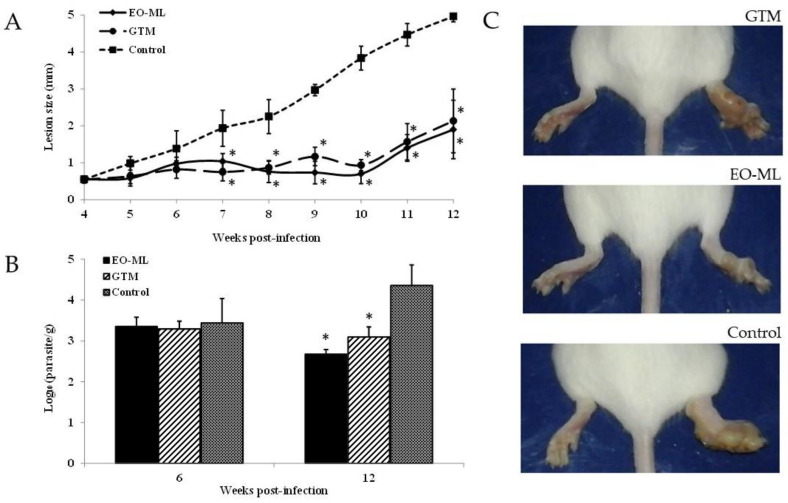
Antileishmanial effect of the essential oil from *Melaleuca leucadendra* collected in National Botanic Garden, Havana, Cuba, on BALB/c mice infected with 5 × 10^6^ promastigotes of *L. amazonensis*/animal. Treatment started four weeks post-infection with essential oil from *Melaleuca leucadendra* or Glucantime^®^ with five doses by the intralesional route at 30 mg/kg every four days. (**A**): Lesion size; (**B**): Parasite burden; (**C**): Pictures at 12 weeks post-infection of infected animals in the footpad with *L. amazonensis* and treated. EO-ML: essential oil from *M. leucadendra*; GTM: Glucantime^®^ used as reference drug; Control: Untreated animals. *: Displays statistical differences (*p* < 0.05) compared to control untreated animals.

In the in-vivo model, the studied oil was able to control the disease progression, which was shown in the statistically significant smaller (*p* < 0.05) lesion size (Figure 3A) and parasite burden (Figure 3B) with respect to untreated animals. Compared with Glucantime^®^ (GTM), a similar efficacy (*p* > 0.05) was found for EO-ML, as shown for lesion size (Figure 3A) and parasite burden (Figure 3B). Figure 3C shows the differences among cutaneous lesions for each group, which was represented by one animal selected at random.

Here, we report the in vivo efficacy of EO-ML against *L. amazonensis* infection by using 30 mg/kg for 15 days, with a four-day interval by the subcutaneous route, for a total of five doses. In other studies, however, higher numbers of doses and concentrations of antileishmanial products were usually employed in the animals, aiming to enhance their efficacy. For example, oil from the trunk of *Copaifera martii* Hayne was administered at 100 mg/kg/day for 30 days [39]; while the treatment with the EO from *Carapa guianensis* Aubl. Was performed for eight weeks using 100–160 mg/kg/day [40].

Our data show the efficacy of EO-ML to be comparable with animals treated with GTM, a first-line drug, taking into account the evaluated parameters (dosing, lesion size, and parasite burden). The significant effect of EO-ML on the reduction of BALB/c mice infection caused by *L. amazonensis* is indicative of a reduced pathology; a complete cure was not observed, however.

The positive effect observed in animals treated with EO-ML, could be explained by the direct activity of the oil on the growth of the parasite, as shown in the in-vitro assays. Nevertheless, the efficacy exhibited in the murine model could also be associated with the ability of oil constituents to induce indirect effects that can contribute to controlling a *Leishmania* infection in the treated animals. Recent studies have indicated that terpinen-4-ol and limonene present anti-inflammatory activity, β-pinene has shown antioxidant effect [41], and α-pinene modulated macrophage activation by the stimulation of NO production and increasing the phagocytic and lysosomal activities [42]. All these pharmacological properties could contribute to controlling a leishmaniasis infection.

## 3. Materials and Methods

### 3.1. Plant and Essential Oils

Aerial parts (leaves and stems) of *M. leucadendra* plant were collected during early hours of the morning in March 2015 at National Botany Garden (NBG), Havana, Cuba, and a specimen was deposited at the Herbarium of Cuban Flora of the NBG and authenticated by M.Sc. Eldys Bécquer (voucher number: 8501918). Leaves of *M. leucadendra* were manually selected, rinsed with abundant water, and crushed into small pieces. Immediately, the fresh vegetal material was conventionally hydrodistilled using a Clevenger-type apparatus for 5 h, and the EO-ML was obtained, yielding 0.8%. The essential oil was stored in a sealed amber vial at 4 °C until analysis and screening.

A sample of 100 µL was used to carry out the chemical characterization by GC-MS using a Shimadzu GCMS-QP2010 Ultra (Shimadzu Scientific Instruments, Columbia, MD, USA). A 5% *w/v* solution of the sample in CH_2_Cl_2_ was prepared and 0.1 μL was injected with a splitting mode (30:1). In this case, a ZB-5 fused silica capillary column with (5% phenyl)-polymethylsiloxane as stationary phase (film thickness of 0.25 μm, a length of 30 m, and an internal diameter of 0.25 mm (Phenomenex, Torrance, CA, USA) and the carrier gas was helium (column head pressure of 552 kPa, flow rate of 1.37 mL/min, injector temperature of 250 °C and an ion source temperature of 200 °C) were used. Equipment operated in the electron impact (EI) mode (electron energy = 70 eV), scan range = 40–400 atomic mass units, scan rate = 3.0 scans/s and GC-MS solution software. The GC oven temperature program was set to 50 °C as the initial temperature, which increased at 2 °C/min until 260 °C. Finally, identification of the oil components was based on their retention indices (RI) determined by reference to a homologous series of *n*-alkanes, and by comparison of their mass spectral fragmentation patterns with those reported in the literature [43], and stored in our in-house Sat-Set library [44].

Another aliquot of EO-ML and 1,8-cineole (purity 99%; Sigma-Aldrich, St. Louis, MO, USA) were used to carry out the biological assays. The EO-MS and 1,8-cineole were dissolved in dimethylsulfoxide (DMSO; BDH, Poole, England) at 20 mg/mL and 40 mM, respectively.

### 3.2. Antimicrobial Assays

The screening of antimicrobial activity of EO was performed on a panel of reference strains and clinical isolates (obtained from the Collections of State Scientific Center of Antibiotics for antimicrobial activity): *S. aureus* ATCC 29213, *E. faecalis* ATCC 29212, *E. coli* ATCC 25922, *P. aeruginosa* ATCC 27853, *C. parapsilosis* ATCC, *A. niger* 37a and *M. canis* B-200. The MIC values were determined by the broth micro-dilution method using Mueller Hinton broth and National Committee for Clinical Laboratory Standards procedures.

### 3.3. Antikinetoplastid Assays

For antitrypanosomal activity, two species were included: *T. brucei* (Squib-427) and *T. cruzi* (Tulahuen CL2). For *T. brucei*, 1.5 × 10^4^ trypomastigotes cultured in Hirumi-9 medium supplemented with 10% inactivated fetal calf serum (FCSi; Invitrogen, Belgium) and tested products were added in a 96-well plate and incubated at 37 °C and 5% CO_2_ for 72 h [45]. Then, parasite growth was assessed fluorimetrically using 20 µL of resazurin (Sigma-Aldrich, St. Louis, MO, USA) at 50 µg/mL. The plate was incubated for an additional 24 h under the same conditions and read at 530 nm excitation and 590 nm emission in a Tecan GENios Multifunction Fluorimeter (Tecan Group, Maennedorf, Switzerland). On the other hand, EO at different concentrations was added to 4 × 10^4^ amastigotes in 4 × 10^3^ MRC-5 cells using minimal essential medium (MEM; Life Technologies, Carlsbad, CA, USA) supplemented with 20 mM l-glutamine, 16.5 mM sodium bicarbonate, and 5% of FCSi in a plate of 96-wells and incubated for seven days at 37 °C and 5% CO_2_. In this case, parasite viability was determined colorimetrically by adding the β-galactosidase substrate chlorophenol red β-d-galactopyranoside (Sigma Aldrich, St. Louis, MO, USA). Finally, the absorbance was read at 540 nm after 4 h of incubation at 37 °C [46].

For antileishmanial activity, the intracellular amastigote model of *L. amazonensis* (MHOM/77BR/LTB0016) was used. PMM were obtained by peritoneal washing with RPMI medium (Sigma, St. Louis, MO, USA) supplemented with antibiotics from healthy BALB/c mice and plated at 10^6^/mL in a 24-well plate. After incubation at 37 °C and 5% CO_2_ for 2 h, non-adherent cells were removed, and stationary-phase promastigotes were added at a 4:1 parasite/macrophage ratio in the medium supplemented with heat-inactivated fetal bovine serum (HFBS; Sigma-Aldrich, St. Louis, MO, USA). The plate was incubated at the same condition for 4 h, and free parasites were also removed. Subsequently, products were added, and four serial dilutions were carried out. The plate was incubated at the same conditions for 48 h as described above [47]. After that, the supernatant was discarded, cells were fixed with methanol, stained with 10% Giemsa and microscopically examined (Motic, Japan) under immersion oil at 1000×. The total parasite burden was determined according to the number of infected macrophages and the number of amastigotes inside the macrophages after counting of 100 macrophages.

### 3.4. Antiproliferative and Cytotoxicity Screening on Malignant and Non-Malignant Cells

To determine the activity of the EO on malignant cells, the following cancer cell lines were used: (i) 22Rv1 (human prostate carcinoma, ATCC^®^ CRL-2505TM) cultivated in RPMI-1640 medium (Gibco-Life Technologies, Paisley, UK) supplemented with RPMI-1640 Vitamins (PanEco, Moscow, Russia), (ii) MCF-7 (human breast cancer, ATCC^®^ HTB-22) cells, as well (iii) MCF-7/Rap and (iv) MCF-7/4OHTAMO resistant sublines (The rapamycin-resistant MCF-7/Rap and 4OH-tamoxifen-resistant MCF-7/4OHTAMO sublines were established from the parent MCF-7 cells by long-term rapamycin or 4OH-tamoxifen treatment, respectively), and (v) EFO-21 (ovary cystadenocarcinoma, DSMZ ACC 235) cultured in standard 4.5 g/L glucose DMEM medium (Gibco-Life Technologies, Paisley, UK). In all cases, culture was supplemented with 10% FCSi, antibiotics (50 μg of streptomycin/mL and 50 U of penicillin/mL) and 0.1 mg/mL sodium pyruvate (Santa Cruz Biotechnology, Dallas, TX, USA) and maintained in a NuAir incubator (NuAir, Plymouth, MN, USA) at 37 °C, 5% CO_2_ and 80–85% humidity. Then, 100 × 10^3^ 22Rv1 cells/well, 40 × 10^3^ MCF-7, MCF-7/Rap or MCF-7/4OHTAMO cells/well, and 110 × 10^3^ EFO-21 cells/well were seeded into 24-well plates in 900 μL of the medium, and the plates were incubated for 24 h at 37 °C and 5% CO_2_. Follow, different concentrations of EO were added and the plates were incubated for 72 h under the same conditions, which the cellular viability was assessed using the 3-[4,5-dimethylthiazol-2-yl]-2,5-diphenyltetrazolium bromide (MTT; AppliChem GmbH, Darmstadt, Germany) at 0.2 mg/mL per well [48,49]. After additional incubation of 2 h, the supernatant was discarded, the MTT formazan purple crystals were dissolved in DMSO (350 μL per well), and the absorbance was measured at 571 nm and 630 nm as a reference in a MultiScan reader (ThermoFisher, Waltham, MA, USA) after the plates were gently shaken.

Cytotoxicity on non-malignant cells was also studied using three models: (i) MCF-10A (ATCC^®^ CRL-10317) normal breast cells cultured in DMEM/F12 (Gibco) supplemented with 5% donor horse serum (BioSera), 20 ng/mL EGF (PanEco), 0.5 µg/mL hydrocortisone (ChemCruz), and 10 µg/mL insulin (PanEco) at 37 °C, 5% CO_2_ and 80–85% humidity), (ii) J774A.1 (murine macrophage cell line, ATCC^®^, TIB-67™) cultivated in Dulbecco’s modified eagle medium (DMEM; Thermo Fisher Scientific, Waltham, MA, USA) supplemented at 10% of HFBS, antibiotics and maintained on a roller culture apparatus at 5 rpm and (iii) PMM isolated with RPMI and antibiotics at the moment of use. Briefly, 50 × 10^3^ MCF-10A cells were seeded into 24-well plates in 900 μL of the medium, and then, the activity of EO-ML was tested as described above for the case of MCF-7 breast cancer cells. In the case of J774A.1 cells, 200 µL with 10^5^ cells/mL were distributed in the wells of the 96-well microplates, incubated at 37 °C, 5% CO_2_ for 24 h to allow attachment, and non-adherent cells were eliminated after washing. Then, fresh medium and different concentrations of EO were added, and the plate was incubated for an additional 24 h at the same condition. Finally, cellular viability was determined fluorometrically by the resazurin method, as previously described [50]. In the case of PMM, macrophages were obtained by peritoneal washing in RPMI medium and antibiotics as mentioned and seeded at 3 × 10^5^ cells/mL. The plate was incubated at 37 °C, and 5% CO_2_ for 2 h, and non-attached cells were removed. Then, fresh medium with HFBS and EO at different concentrations were added to additional incubation for 48 h under the same conditions. Cellular viability was then measured by the MTT method as described above.

### 3.5. In Vivo Evaluation on Cutaneous Leishmaniasis Caused by L. amazonensis

Female healthy BALB/c mice were infected in the right hind footpad with 5 × 10^6^ stationary-phase promastigotes of L. amazonensis by the subcutaneous route, which was identified as day 0. Then, four weeks p.i., the animals were randomly distributed into three groups of eight mice each, and two groups of mice were treated with EO-ML or GTM at 30 mg/kg. Products were applied every four days to a total of five doses by the intralesional route. The third group of animals was considered to be the control group that received no treatment. In parallel, from four weeks p.i. until 12 weeks p.i, the animals were daily observed, body weight and the lesion size were weekly supervised using a technical bascule (SCALTEC, Göttingen, Germany) and a caliper to measure footpad swelling and lesion diameter, respectively. In these cases, a variation of body weight, as well as an average of lesion size (mean of the differences between infected and uninfected footpads), was calculated with respect to week 4 p.i. for each group. In addition, the parasite burden was determined on weeks 6 and 12 p.i. through the culture microtitration method in 96-well plates [51]. Briefly, three animals selected at random from each group were killed by cervical dislocation, a sample of subcutaneous tissues from the infected area was excised, weighed, and homogenized in 4 mL of Schneider’s medium. Then, a four-fold serial dilution was carried out in a 96-well plates in duplicate under sterile conditions and incubated at 26 °C. After seven days of incubation, the plates were examined under an inverted microscope (Olympus, Tokyo, Japan) at 400× to select the last dilution that contained at least one mobile parasite, which was defined as the final titer. The parasite burden was calculated as the geometric mean of reciprocal titers/weight of tissue sample multiplied by 400. All of the experimental procedures involving animals were conducted in accordance with the Guide for the Care and Use of Laboratory Animals, Eighth Edition, which was approved by the Ethics Committee (CEI-IPK 14-12), Havana, Cuba.

### 3.6. Statistical Analysis

For in vitro assays, the IC_50_ for each product on each system was obtained from dose-response curves, results were expressed as mean ± SD of three replicates, and comparisons among values were performed using Mann-Whitney test with Statistica for Windows Program (Version 10, StatSoft, Inc., Tulsa, OK, USA). For in vivo experiments, lesion evolution and parasite load were processed by the Variance Analysis Test (ANOVA), followed by a Post Hoc Test (LDS test or planned comparison). In all cases, statistically significant differences were identified for *p* < 0.05.

## 4. Conclusions

To the best of our knowledge, this is the first report on antiproliferative potentialities of EO-ML growing in Cuba, confirming the medical value of the plant. However, the inability of 1,8-cineole to demonstrate a strong antikinetoplastid activity reveals that other ingredients or a possible synergism are involved in the activity of the EO. In particular, the leishmanicidal effect of EO-ML was confirmed in vivo using a murine model of CL, which the tested product was able to significantly reduce the lesion size and parasite burden in infected tissues similar to the reference drug.

In addition, our results open new perspectives to further investigations to clarify: (i) Therapeutic value of EO-ML for African trypanosomiasis on relevant *T. brucei rhodesiense* and *T. brucei gambiense* infectious agents through in vitro and in vivo models, (ii) the possibility of the application of this oil as alternative to traditional treatment in cancer-resistant cells, (iii) potentialities of a combination of oil constituents, and (iv) mechanism of action of EO-ML, as well as the pure compounds. In conclusion, the results reported here represent an advancement in studies on EOs as drug candidates and might promote preclinical investigations on oil standardization.

## Figures and Tables

**Table 1:** Peak assignment for gas chromatography-mass spectrometry profiles of the essential oil extracted by hydrodistillation from *Melaleuca leucadendra* collected in National Botanic Garden, Havana, Cuba.

RI	Compound	%	RI	Compound	%
858	(3*Z*)-Hexen-1-ol	0.1	1167	Borneol	0.1
928	α-Thujene	tr	1169	δ-Terpineol	0.3
**934**	**α-Pinene**	**2.7**	1174	Ethyl benzoate	tr
947	Camphene	0.1	**1179**	**Terpinen-4-ol**	**1.2**
957	Benzaldehyde	0.1	**1194**	**α-Terpineol**	**15.6**
**973**	**β-Pinene**	**1.2**	1200	Methyl chavicol (=Estragole)	0.1
989	Myrcene	0.5	1230	Citronellol	0.1
1001	α-Phellandrene	0.1	1235	Ascaridole	tr
1007	δ-3-Carene	0.1	1277	Safrole	tr
1014	α-Terpinene	tr	1280	Unidentified	0.1
1023	*p*-Cymene	0.7	1420	β-Caryophyllene	0.2
**1027**	**Limonene**	**4.8**	1451	α-Humulene	tr
**1031**	**1,8-Cineole**	**61.0**	1458	*allo*-Aromadendrene	tr
1049	(*E*)-β-Ocimene	tr	1492	Viridiflorene (=Ledene)	tr
1059	γ-Terpinene	0.2	1563	Palustrol	0.1
1090	Terpinolene	0.1	**1589**	**Viridiflorol**	**7.9**
1096	Methyl benzoate	0.1	1597	Guaiol	0.2
1103	Linalool	0.2	1600	Ledol	0.8
1114	*endo*-Fenchol	0.1	1606	Humulene epoxide II	0.1
1147	*neo*-Isopulegol	0.4	1642	τ-Cadinol	0.1
1149	Camphene hydrate	tr	1650	β-Eudesmol	0.1
1158	*iso*-Isopulegol	0.1	1653	α-Eudesmol	0.1

RI: Retention Index (determined with respect to a homologous series of *n*-alkanes on a ZB-5 column). Tr: Trace (concentration < 0.05%). Major components (>1%) are highlighted in **bold**.
